# Classification and sensitivity analysis of the transmission dynamic of hepatitis B

**DOI:** 10.1186/s12976-017-0068-3

**Published:** 2017-12-05

**Authors:** Tahir Khan, Il Hyo Jung, Amir Khan, Gul Zaman

**Affiliations:** 1 Department of Mathematics, University of Malakand, Khyber Pakhtunkhawa, Chakdara Dir Lower, Pakistan; 2 Department of Mathematics, Pusan National University, Busan, 46241 South Korea; 3 Department of Mathematics, University of Swat, Mingora, Swat, Khyber Pakhtunkhawa Pakistan

**Keywords:** Hepatitis B epidemic model, Basic reproduction number, Stability analysis, Lyapunov function theory, Geometrical approach, Numerical simulation

## Abstract

**Background:**

Hepatitis B infection caused by the hepatitis B virus is one of the most serious viral infections and a global health problem. In the transmission of hepatitis B infection, three different phases, i.e. acute infected, chronically infected, and carrier individuals, play important roles. Carrier individuals are especially significant, because they do not exhibit any symptoms and are able to transmit the infection. Here we assessed the transmissibility associated with different infection stages of hepatitis B and generated an epidemic model.

**Methods:**

To demonstrate the transmission dynamic of hepatitis B, we investigate an epidemic model by dividing the infectious class into three subclasses, namely acute infected, chronically infected, and carrier individuals with both horizontal and vertical transmission.

**Results:**

Numerical results and sensitivity analysis of some important parameters are presented to show that the proportion of births without successful vaccination, perinatally infected individuals, and direct contact rate are highest risk factors for the spread of hepatitis B in the community.

**Conclusion:**

Our work provides a coherent platform for studying the full dynamics of hepatitis B and an effective direction for theoretical work.

## Background

Hepatitis implies the inflammation of liver. Hepatitis B infection caused by the hepatitis B virus is among the most serious viral infections. It is a global health problem and one of the leading causes of death around the world. Worldwide, 2 billion people are infected with hepatitis B virus and about 360 million individuals live with chronic hepatitis B infection [1, 2]. In addition, hepatitis B virus infection is responsible for about 80% of primary liver cancers [3]. Therefore, every year approximately 780,000 individuals die from chronic or acute hepatitis B virus infection [1]. Hepatitis B virus can be transmitted from one individual to another in different ways, such as transmission through blood (sharing of razors, blades, or toothbrushes), semen, and vaginal secretions (unprotected sexual contact) [4–7]. The other major transmission route is from an infected mother to her child during childbirth, which is called vertical transmission. However, hepatitis B virus cannot be transmitted through water, food, hugging, kissing, or causal contact such as in the work place, school, etc. [6–8].

Hepatitis B infection has multiple phases: acute, chronic, and carrier. Acute hepatitis B is a short-term infection within the first 6 months after someone is infected with the virus. In this stage, the immune system is usually able to clear the virus from the body, and recover within a few months. Chronic hepatitis B refers to the illness that occurs when the virus remains in the individual’s body and, over time, the infection develops into a serious health problem. Individuals with chronic hepatitis often have no history of acute illness; however, it can cause liver scarring, which becomes the cause of liver failure and may also develop into liver cancer [3]. The phase at which the individuals do not exhibit any symptoms, but transmit the disease to others is known as the carrier phase, which plays an important role in the transmission of hepatitis B infection. This is the most dangerous and serious phase of hepatitis B, because it is difficult to control the hepatitis B virus infection when a large group of carriers exist, as they will be responsible for transmitting the disease to new individuals.

Mathematical modeling is a powerful tool to describe the dynamical behavior of different diseases in the real world [9, 10]. Several mathematicians and biologists have developed different epidemic models to understand and control the spread of transmissible diseases in the population. In the last two decades, the field of mathematical modeling has been used frequently for the study of transmission of different types of infectious diseases. Mann and Roberts [3] and Thornley et al. [8] used a mathematical model for eliminating hepatitis B virus in New Zealand. In 1991, Anderson and May [11] described the effect of carriers on the transmission of hepatitis B virus by using a simple deterministic model. Zhao et al. [12] presented an age structured model for the prediction of the dynamics of hepatitis B virus transmission and evaluated the long-term effectiveness of the vaccination program in China. In 2010, Zou et al. [13] presented a model for the transmission dynamics and control of the hepatitis B virus in China. Recently, a mathematical model for the transmission dynamics and optimal control of hepatitis B has been presented by Khan et al. [14].

The different phases of hepatitis B play a very important role in the transmission of hepatitis B infection, and have not yet been investigated collectively for their potential role in generating a hepatitis B epidemic model. We consider a hepatitis B epidemic model by identifying the different phases, acute, chronic, and carrier, of hepatitis B infection.

## Methods

With the different stages of hepatitis B, the total population is classified into seven different compartments with three infectious epidemiological classes, namely acute infected, chronically infected, and carrier individuals. First we develop the model, then we investigate the equilibria. For a biologically feasible region, we show the boundedness. Further, we find the basic reproduction number by using the next-generation matrix approach. In addition, we prove the local and global asymptotic stability of the proposed model. For the local stability, we use the method of linearization and Routh–Herwitz criteria. The global asymptotic stability is retrieved by using the method proposed by Castillo-Chávez et al. [15] and a geometrical approach. Finally, the numerical simulations are carried out by using a fourth-order Runge–Kutta method to show the feasibility of the obtained results. Moreover, a sensitivity analysis of some important parameters is also presented. Our work provides a coherent platform for studying the full dynamics of hepatitis B and an effective direction for theoretical work. In view of the characteristics of hepatitis B, we develop an epidemic model of hepatitis B by dividing the total population into seven epidemiological subclasses: susceptible *S*(*t*), latent *L*(*t*), acute infected *A*(*t*), chronic infected *B*(*t*), carrier *C*(*t*), recovered with permanent immunity *R*(*t*), and vaccinated *V*(*t*). We place the following assumptions on the model.

- *A*_*1*_ The initial populations *S*(0), *L*(0), *A*(0), *B*(0),*C*(0), *V*(0), and *R*(0) are all known and non-negative.
- *A*_*2*_ Recovered individuals have permanent immunity.
- *A*_*3*_ The inflow of newborns with successful vaccination go into the vaccinated subclass.
- *A*_*4*_ The inflow of newborns with perinatal infection go into the carrier subclass.
- *A*_*5*_ The inflow of newborns without perinatal infection go into the susceptible subclass.
- *A*_*6*_ The population with successful vaccination go into the vaccinated subclass.

Thus, the mathematical model can be presented by the following system of seven ordinary differential equations,

1 $$ {\displaystyle \begin{array}{ll}\frac{dS(t)}{dt}\hfill & = b\xi \left(1-\eta C(t)\right)+\varphi V(t)-\beta A(t)S(t)-\gamma \beta B(t)S(t)-\zeta \beta C(t)S(t)\hfill \\ {}\hfill & -\left({\mu}_0+v\right)S(t),\hfill \\ {}\frac{dL(t)}{dt}\hfill & =\beta S(t)A(t)+\gamma \beta S(t)B(t)+\zeta \beta S(t)C(t)-\left(\sigma +{\mu}_0\right)L(t),\hfill \\ {}\frac{dA(t)}{dt}\hfill & =\sigma L(t)-\left({\mu}_0+{\gamma}_1+\psi \right)A(t),\hfill \\ {}\frac{dB(t)}{dt}\hfill & =p{\gamma}_1A(t)-\left({\mu}_0+{\mu}_1+{\gamma}_2\right)B(t),\hfill \\ {}\frac{dC(t)}{dt}\hfill & = b\xi \eta C(t)+\left(1-p\right){\gamma}_1A(t)-\left({\mu}_0+{\mu}_2+{\gamma}_3\right)C(t),\hfill \\ {}\frac{dR(t)}{dt}\hfill & =\psi A(t)+{\gamma}_2B(t)+{\gamma}_3C(t)-{\mu}_0R(t),\hfill \\ {}\frac{dV(t)}{dt}\hfill & =b\left(1-\xi \right)+ vS(t)-\left({\mu}_0+\varphi \right)V(t).\hfill \end{array}} $$

In the model (1), *b* represents the birth rate, *ξ* represents the proportion of births without successful vaccination, *η* represents the proportion of perinatally infected individuals, *φ* represents the rate of waning vaccine-induced immunity, *β* represents the transmission rate from susceptible to infected, *γ* and *ζ* represent the reduced transmission rate of chronic and carrier individuals infected with hepatitis B, respectively. The natural death rate is represented by *μ*_0_. We use *v* to denote the vaccination rate, *σ* represents the moving rate from latent class to acute class, *γ*_1_ represents the moving rate from acute to chronic and carrier, *ψ* represents the recovery rate from acute class to recovered, *γ*_2_ represents the moving rate of chronic carrier to immune, *γ*_3_ represents the moving rate of carrier to immune, *μ*_1_ and *μ*_2_ represent the death rates occurring from hepatitis B, and *p* represents the average probability of an individual’s failure to clear an acute infection and going to the carrier state.

To represent the dynamics of our proposed model (1), we need to find the equilibria of the proposed model (1), which are disease-free and endemic equilibria.

### Equilibrium analysis

The disease-free equilibrium point of the model (1) is denoted by *E*_0_ and defined as *E*_0_ = (*S*_0_, 0, 0, 0, 0, 0, *V*_0_), where

2 $$ {S}_0=\frac{b\left(\varphi +{\mu}_0\xi \right)}{\mu_0\left({\mu}_0+v+\varphi \right)},\kern1em {V}_0=\frac{b\left({\mu}_0+v-{\mu}_0\xi \right)}{\mu_0\left({\mu}_0+v+\varphi \right)}. $$

Similarly, the endemic equilibrium point is denoted by *E*_1_ = (*S*_1_, *L*_1_, *A*_1_, *B*_1_, *C*_1_, *R*_1_, *V*_1_), where

3 $$ {\displaystyle \begin{array}{ll}{S}_1\hfill & =\frac{S_0}{R_0},\hfill \\ {}{L}_1\hfill & =\frac{S_1}{\left(\sigma +{\mu}_0\right)}\left( b\xi +\varphi {V}_1-{S}_1\left({\mu}_0+v\right)\right),\hfill \\ {}{A}_1\hfill & =\frac{\sigma {L}_1}{\gamma_1+\psi +{\mu}_0},\hfill \\ {}{B}_1\hfill & =\frac{\sigma p{\gamma}_1{L}_1}{\left({\gamma}_2+{\mu}_0+{\mu}_1\right)\left({\gamma}_1+\psi +{\mu}_0\right)},\hfill \\ {}{C}_1\hfill & =\frac{{\sigma \gamma}_1\left(1-p\right){L}_1}{\left({\gamma}_1+\psi +{\mu}_0\right)\left({\gamma}_3+{\mu}_0+{\mu}_1- b\eta \xi \right)},\hfill \\ {}{R}_1\hfill & =\frac{1}{\mu_0}\left(\psi {A}_1+{\gamma}_2{B}_1+{\gamma}_3{C}_1\right),\hfill \\ {}{V}_1\hfill & =\frac{b\left(1-\xi \right)}{\varphi +{\mu}_0}+{vS}_1.\hfill \end{array}} $$

### Boundedness

For the biologically feasible region, we prove the boundedness of the proposed model.

**Theorem 1** *The solution of the model (1) is bounded.*

*Proof*: Let *N*(*t*) denote the total population, then *N*(*t*) = *S*(*t*) + *L*(*t*) + *A*(*t*) + *B*(*t*) + *C*(*t*) + *R*(*t*) + *V*(*t*). Differentiation of *N*(*t*) with respect to time and the use of model (1) yields $\frac{dN(t)}{dt}= b\xi -{\mu}_0N(t)-{\mu}_1B(t)-{\mu}_2C(t)$. Therefore, we can write $\frac{dN(t)}{dt}+{\mu}_0N(t)\le b\xi .$ Integrating both sides and then using the theory of differential inequality [16], we obtain $0<N\left(S,L,A,B,C,R,V\right)\le \frac{b\xi}{\mu_0}\left(1-{e}^{-{\mu}_0t}\right)+{N}_0{e}^{-{\mu}_0t}.$ Now let *t* → ∞, it becomes $0<N\left(S,L,A,B,C,R,V\right)\le \frac{b\xi}{\mu_0}.$ Hence, the solution of the model (1) initiating in ${R}_{+}^7$ is limited in the set $\varDelta =\left\{\left(S,L,A,B,C,R,V\right)\in {R}_{+}^7:N=\frac{b\xi}{\mu_0}+\xi \right\}$ for any *ξ* > 0 and *t* →  ∞ , which completes the proof.

### Basic reproduction number

The threshold quantity that determines whether an epidemic arises or the infection dies out is called the basic reproduction number of the disease, which is a key concept [11, 17]. It represents the expected average number of new infections produced directly and indirectly by a single infected individual, when introduced into a completely susceptible population. To find the basic reproduction number for the proposed model (1), we use the method of Driessche and Watmough [18]. Let *χ* = (*L*(*t*), *A*(*t*), *B*(*t*), *C*(*t*))^*T*^, so from the model (1), we have

4 $$ \frac{d\chi}{d t}=\overline{F}-\overline{V}. $$

In eq. (4), $\overline{F}$ and $\overline{V}$ are the matrices that contain the nonlinear and linear terms, respectively, such that

$$ \overline{F}=\left(\begin{array}{c}\hfill \beta S(t)A(t)+\gamma \beta S(t)B(t)+\xi \beta S(t)C(t)\hfill \\ {}\hfill 0\hfill \\ {}\hfill 0\hfill \\ {}\hfill 0\hfill \end{array}\right), $$

$$ \overline{V}=\left(\begin{array}{c}\hfill \left(\sigma +{\mu}_0\right)L(t)\hfill \\ {}\hfill \left({\mu}_0+{\gamma}_1+\psi \right)A(t)-\sigma L(t)\hfill \\ {}\hfill \left({\mu}_0+{\mu}_1+{\gamma}_2\right)B(t)-p{\gamma}_1A(t)\hfill \\ {}\hfill \left({\mu}_0+{\mu}_2+{\gamma}_3\right)C(t)- b\xi \eta C(t)-\left(1-p\right){\gamma}_1A(t)\hfill \end{array}\right). $$

Now, we find the Jacobian matrix of $\overline{F}$ and $\overline{V}$ at the disease-free equilibrium *E*_0_, which becomes

$$ F= Jacobian of\ \overline{F}\  at\  DFE=\left(\begin{array}{cccc}\hfill 0\hfill & \hfill \beta {S}_0\hfill & \hfill \gamma \beta {S}_0\hfill & \hfill \zeta \beta {S}_0\hfill \\ {}\hfill 0\hfill & \hfill 0\hfill & \hfill 0\hfill & \hfill 0\hfill \\ {}\hfill 0\hfill & \hfill 0\hfill & \hfill 0\hfill & \hfill 0\hfill \\ {}\hfill 0\hfill & \hfill 0\hfill & \hfill 0\hfill & \hfill 0\hfill \end{array}\right), $$

$$ V= Jacobian of\ \overline{V}\  at\  DFE=\left(\begin{array}{cccc}\hfill {a}_{11}\hfill & \hfill 0\hfill & \hfill 0\hfill & \hfill 0\hfill \\ {}\hfill -\sigma \hfill & \hfill {a}_{22}\hfill & \hfill 0\hfill & \hfill 0\hfill \\ {}\hfill 0\hfill & \hfill -{a}_{32}\hfill & \hfill {a}_{33}\hfill & \hfill 0\hfill \\ {}\hfill 0\hfill & \hfill -{a}_{41}\hfill & \hfill 0\hfill & \hfill {a}_{44}\hfill \end{array}\right), $$

where *a*_11_ = *σ* + *μ*_0_, *a*_22_ = *μ*_0_ + *γ*_1_ + *ψ*, *a*_32_ = *pγ*_1_, *a*_33_ = *μ*_0_ + *μ*_1_ + *γ*_2_, *a*_41_ = (1 − *p*)*γ*_1_, and *a*_44_ = *μ*_0_ + *μ*_0_ + *γ*_3_ − *bξη*. Thus, the basic reproduction number *R*_0_ is the spectral radius of the next-generation matrix $\overline{K}={FV}^{-1},$ that is, ${R}_0=\rho \left(\overline{K}\right)=\rho \left({FV}^{-1}\right)=\max \left\{|{\lambda}_1|,\dots, |{\lambda}_4|\right\},$ where *λ*_*i*_ for *i* = 1, 2, 3, 4 are the eigenvalues of $\overline{K}$. Hence, the basic reproduction number *R*_0_ for our proposed model (1) becomes

5 $$ {R}_0={\overline{R}}_1+{\overline{R}}_2+{\overline{R}}_3, $$

where

$$ {\displaystyle \begin{array}{lll}{\overline{R}}_1\hfill & =\frac{\sigma \beta {S}_0}{\left(\sigma +{\mu}_0\right)\left({\gamma}_1+\psi +{\mu}_0\right)},\hfill & \hfill \\ {}{\overline{R}}_2\hfill & =\frac{{\sigma \beta \gamma \gamma}_1{pS}_0}{\left(\sigma +{\mu}_0\right)\left({\gamma}_1+\psi +{\mu}_0\right)\left({\gamma}_2+{\mu}_0+{\mu}_1\right)},\kern1em {\overline{R}}_3\hfill & =\frac{{\sigma \beta \zeta \gamma}_1\left(1-p\right){S}_0}{\left(\sigma +{\mu}_0\right)\left({\gamma}_1+\psi +{\mu}_0\right)\left({\gamma}_3+{\mu}_0+{\mu}_2- b\xi \eta \right)}.\hfill \end{array}} $$

### Local stability analysis

In this subsection, we discuss the local asymptotic satiability of the proposed model (1) at disease-free equilibrium *E*_0_ and endemic equilibrium *E*_1_. To show the local asymptotic stability, we reduce the proposed model, because *R* appears only in the sixth equation of the model. Thus, the reduced model is given by

6 $$ {\displaystyle \begin{array}{ll}\frac{dS(t)}{dt}\hfill & = b\xi \left(1-\eta C(t)\right)+\varphi V(t)-\beta A(t)S(t)-\gamma \beta B(t)S(t)-\zeta \beta C(t)S(t)\hfill \\ {}\hfill & -\left({\mu}_0+v\right)S(t),\hfill \\ {}\frac{dL(t)}{dt}\hfill & =\beta S(t)A(t)+\gamma \beta S(t)B(t)+\zeta \beta S(t)C(t)-\left(\sigma +{\mu}_0\right)L(t),\hfill \\ {}\frac{dA(t)}{dt}\hfill & =\sigma L(t)-\left({\mu}_0+{\gamma}_1+\psi \right)A(t),\hfill \\ {}\frac{dB(t)}{dt}\hfill & =p{\gamma}_1A(t)-\left({\mu}_0+{\mu}_1+{\gamma}_2\right)B(t),\hfill \\ {}\frac{dC(t)}{dt}\hfill & = b\xi \eta C(t)+\left(1-p\right){\gamma}_1A(t)-\left({\mu}_0+{\mu}_2+{\gamma}_3\right)C(t),\hfill \\ {}\frac{dV(t)}{dt}\hfill & =b\left(1-\xi \right)+ vS(t)-\left({\mu}_0+\varphi \right)V(t).\hfill \end{array}} $$

Regarding the local asymptotic stability of the proposed model at disease-free and endemic equilibrium points, we have the following results.

**Theorem 2** *If R*_0_ > 1*, then the model (1) is locally asymptotically stable at the endemic equilibrium point E*_1_ *, and if R*_0_ < 1*, then it is unstable.*

*Proof:* The Jacobian matrix of model (6) at the endemic equilibrium point *E*_1_ is

7 $$ {J}_1=\left(\begin{array}{cccccc}\hfill -{h}_{11}\hfill & \hfill 0\hfill & \hfill -\beta {S}_1\hfill & \hfill -\gamma \beta {S}_1\hfill & \hfill b\xi \eta -\zeta \beta {S}_1\hfill & \hfill \varphi \hfill \\ {}\hfill {h}_{21}\hfill & \hfill -{h}_{22}\hfill & \hfill \beta {S}_1\hfill & \hfill \gamma \beta {S}_1\hfill & \hfill \zeta \beta {S}_1\hfill & \hfill 0\hfill \\ {}\hfill 0\hfill & \hfill \sigma \hfill & \hfill -{b}_{33}\hfill & \hfill 0\hfill & \hfill 0\hfill & \hfill 0\hfill \\ {}\hfill 0\hfill & \hfill 0\hfill & \hfill p{\gamma}_1\hfill & \hfill {b}_{44}\hfill & \hfill 0\hfill & \hfill 0\hfill \\ {}\hfill 0\hfill & \hfill 0\hfill & \hfill {h}_{53}\hfill & \hfill 0\hfill & \hfill -{h}_{55}\hfill & \hfill 0\hfill \\ {}\hfill v\hfill & \hfill 0\hfill & \hfill 0\hfill & \hfill 0\hfill & \hfill 0\hfill & \hfill -{h}_{66}\hfill \end{array}\right), $$

where *h*_11_ = *βS*_1_ + *γβB*_1_ + *ζβC*_1_ − (*μ*_0_ + *v*), *h*_21_ = *βS*_1_ + *γβB*_1_ + *ζβC*_1_, *h*_22_ = *σ* + *μ*_0_, *h*_33_ = *μ*_0_ + *γ*_1_ + *ψ*, *h*_44_ = *μ*_0_ + *μ*_1_ + *γ*_2_, *h*_53_ = (1 − *p*)*γ*_1_, *h*_55_ = *μ*_0_ + *μ*_2_ + *γ*_3_, *h*_55_ = *μ*_0_ + *μ*_2_ + *γ*_3_ and *h*_66_ = *μ*_0_ + *φ*. Using an elementary row operation to reduce the above matrix to echelon form, we obtain the following matrix

8 $$ {J}_1=\left(\begin{array}{cccccc}\hfill -{K}_{11}\hfill & \hfill 0\hfill & \hfill -\beta {S}_1\hfill & \hfill -\gamma \beta {S}_1\hfill & \hfill b\xi \eta -\zeta \beta {S}_1\hfill & \hfill \varphi \hfill \\ {}\hfill 0\hfill & \hfill -{K}_{22}\hfill & \hfill {K}_{23}\hfill & \hfill {K}_{24}\hfill & \hfill {K}_{25}\hfill & \hfill \varphi {h}_{21}\hfill \\ {}\hfill 0\hfill & \hfill 0\hfill & \hfill {K}_{33}\hfill & \hfill {K}_{34}\hfill & \hfill 0\hfill & \hfill \sigma \varphi {h}_{21}\hfill \\ {}\hfill 0\hfill & \hfill 0\hfill & \hfill 0\hfill & \hfill {K}_{44}\hfill & \hfill {K}_{45}\hfill & \hfill -\sigma \varphi {h}_{21}\hfill \\ {}\hfill 0\hfill & \hfill 0\hfill & \hfill 0\hfill & \hfill 0\hfill & \hfill {K}_{44}\hfill & \hfill \sigma \varphi {h}_{21}\hfill \\ {}\hfill 0\hfill & \hfill 0\hfill & \hfill 0\hfill & \hfill 0\hfill & \hfill 0\hfill & \hfill {K}_{66}\hfill \end{array}\right), $$

where *K*_11_ =  − *h*_11_, *K*_22_ = *h*_11_ *h*_22_, *K*_23_ = *βS*_1_(*h*_11_ − *h*_21_), *K*_24_ = *γβS*_1_(*h*_11_ − *h*_21_), *K*_25_ = *ζβS*_1_(*h*_11_ − *h*_21_), *K*_33_ =  − *h*_11_ *h*_22_ *h*_33_ + *σβS*_1_(*h*_11_ − *h*_21_), *K*_34_ = *σγβS*_1_(*h*_11_ − *h*_21_), *K*_35_ = *σζβS*_1_(*h*_11_ − *h*_21_), ${K}_{44}=-\frac{\sigma \beta {S}_1{h}_{44}}{p{\gamma}_1}\left(\left({h}_{11}-{h}_{21}\right)+{h}_{11}{h}_{22{h}_{33}}\right),$ *K*_45_ = *ζβS*_1_(*h*_11_ − *h*_21_), *K*_55_ = *K*_44_ + *σζβS*_1_(*h*_11_ − *h*_21_), *K*_66_ = *K*_44_ − *L*, and *L* is defined as

$$ L=\frac{\zeta \beta \left({h}_{11}-{h}_{21}\right)\left(\sigma \varphi {vh}_{44}{h}_{53}\right)}{\beta \left(p{\gamma}_1\zeta {h}_{44}+{h}_{45}\left(p{\gamma \gamma}_1+{h}_{44}\right)\right)}. $$

Thus, the eigenvalues of the Jacobian matrix *J*_1_ are

$$ {\displaystyle \begin{array}{ll}{\lambda}_1\hfill & =-{K}_{11}=-{h}_{11},\kern1em {\lambda}_2=-{K}_{22}=-{h}_{11}{h}_{22},\hfill \\ {}{\lambda}_3\hfill & ={K}_{33}=-{h}_{11}{h}_{22}{h}_{33}+\sigma \beta {S}_1\left({h}_{11}-{h}_{21}\right),\hfill \\ {}{\lambda}_3\hfill & ={K}_{33}=-{h}_{11}{h}_{22}{h}_{33}+\sigma \beta {S}_1\left({h}_{11}-{h}_{21}\right),\hfill \\ {}{\lambda}_4\hfill & ={K}_{44}=-\sigma \beta {S}_1\left({h}_{11}-{h}_{21}\right)\left(\frac{h_{44}}{p{\gamma}_1}+\gamma \right)-\frac{1}{p{\gamma}_1}{h}_{11}{h}_{22}{h}_{33}{h}_{44},\hfill \\ {}{\lambda}_5\hfill & ={K}_{55}=-\frac{1}{p{\gamma}_1}\sigma \beta {S}_1{h}_{44}\left({h}_{11}-{h}_{21}\right)-\frac{1}{p{\gamma}_1}{h}_{11}{h}_{22}{h}_{33}{h}_{44}-\sigma \beta {S}_1\left(\gamma -\zeta \right),\hfill \\ {}{\lambda}_6\hfill & ={K}_{66}={K}_{44}-L.\hfill \end{array}} $$

All eigenvalues except *λ*_5_ have negative real parts and *λ*_5_ is negative, if *γ* > *ζ*. Hence, all eigenvalues of the Jacobian matrix *J*_1_ have negative real part, if *γ* > *ζ*. Therefore, for *R*_0_ > 1, the model (1) is locally asymptotically stable at the endemic equilibrium point *E*_1_, if *γ* > *ζ*.

### Global stability analysis

In this section, the global asymptotic stability of the proposed model for both disease-free as well as at endemic equilibrium is shown. The method of Castillo-Chávez et al. [15] is used to prove the global asymptotic stability at disease-free equilibrium. While to show that the model (1) is globally asymptotically stable at endemic equilibrium, the geometrical approach is implemented, which is a generalization of Lyapunov theory [19]. Here, we give a brief analysis of the Castillo-Chávez et al. method and geometrical approach to prove the global stability of the model (1) at disease-free equilibrium and endemic equilibrium. Thus, by using the method of Castillo-Chávez et al. [15], to the following two subsystems given by

$$ \frac{d{\chi}_1}{dt}=G\left({\chi}_1,{\chi}_2\right), $$

9 $$ \frac{d{\chi}_2}{dt}=H\left({\chi}_1,{\chi}_2\right). $$

In the system (9), *χ*_1_ and *χ*_2_ represent the number of uninfected and infected (latent, acute infected, and chronic carrier) individuals, respectively, that is, *χ*_1_ = (*S*(*t*), *V*(*t*), *R*(*t*)) ∈ *R* ^3^ and *χ*_2_ = (*L*(*t*), *A*(*t*), *B*(*t*), *C*(*t*)) ∈ *R* ^4^. The disease-free equilibrium is denoted by *E*_0_ and defined as ${E}_0=\left({\chi}_1^0,0\right).$ Thus, the existence of global stability at the disease-free equilibrium point depends on the following two conditions.

- If $\frac{{d\chi}_1}{d t}=G\left({\chi}_1,0\right),$ ${\chi}_1^0$ is globally asymptotically stable.
- We have $H\left({\chi}_1,{\chi}_2\right)=B{\chi}_1-\overline{H}\left({\chi}_1,{\chi}_2\right),$ where $\overline{H}\left({\chi}_1,{\chi}_2\right)\ge 0$ for (*χ*_1_, *χ*_2_) ∈ *Δ*..

In the second condition $B={D}_{\chi_2}H\left({\chi}_1^0,0\right)$ is an M-matrix, that is, the off-diagonal entries are positive and *Δ* is the feasible region. Then the following statement holds.

Lemma 1 *For R*_0_ < 1*, the equilibrium point E*_0_ = (*χ* ^0^, 0) *of the system (9) is said to be globally asymptotically stable, if the above conditions are satisfied.*

Similarly, to prove the global stability of the model (1) at endemic equilibrium *E*_1_, we use the geometrical approach [19]. According to this method, we investigate the sufficient condition through which the endemic equilibrium point is globally asymptotically stable. To do this, we consider a system of differential equations given by

10 $$ \dot{x}=f(x), $$

where *f* : *U* → *R* ^*n*^, *U* ⊂ *R* ^*n*^ is an open set simply connected, and *f* ∈ *C* ^1^(*U*). Let us assume that the solution to eq. (10) is *f*(*x* ^∗^) = 0 and for *x*(*t*, *x*_0_), the following hypotheses hold.

- There exists a compact absorbing set *K* ∈ *U*.
- System (10) has a unique equilibrium.

The solution *x* ^∗^ is said to be globally asymptotically stable in *U*, if it is locally asymptotically stable and all trajectories in *U* converge to the equilibrium *x* ^∗^. For *n* ≥ 2, a condition is satisfied for *f*, which precludes the existence of a non-constant periodic solution of eq. (10) known as the Bendixson criterion. The classical Bendixson criterion *divf*(*x*) < 0 for *n* = 2 is robust under *C* ^1^ (see [19]). Further, a point *x*_0_ ∈ *U* is wandering for eq. (10), if there exists a neighborhood *N* of *x*_0_ and *τ* > 0, such that *N* ∩ *x*(*t*, *N*) is empty for all *t* > *τ*. Thus, the following global stability principle is established for an autonomous system in any finite dimension.

Lemma 2 *If conditions* *3* *and* *4* *and the Bendixson criterion are satisfied for eq.* (10)*, then it is robust under C* ^1^ *local perturbation of f at all non-equilibrium, non-wandering points for eq.* (10)*. Then, x* ^∗^ *is globally asymptotically stable in U, provided that it is stable.*

Now to prove the robustness required for Lemma 2, let us define a function, such that

11 $$ P(x)=\left(\begin{array}{c}\hfill n\hfill \\ {}\hfill 2\hfill \end{array}\right)\times \left(\begin{array}{c}\hfill n\hfill \\ {}\hfill 2\hfill \end{array}\right). $$

Eq. (11) is a matrix valued function on *U*. Further, assume that *P* ^−1^ exists and is continuous for *x* ∈ *K*. Now define a quantity, such that

12 $$ \overline{q}=\underset{t\to \infty }{\lim}\sup \kern0.1em \sup \frac{1}{t}{\int}_0^t\left[\mu \left(B\left(x\left(s,{x}_0\right)\right)\right)\right]\kern0.1em ds, $$

where *B* = *P*_*f*_ *P* ^−1^ + *PJ* ^[2]^ *P* ^−1^ and *J* ^[2]^ is the second additive compound matrix of the Jacobian matrix *J*, that is, *J*(*x*) = *Uf*(*x*). Let *ℓ*(*B*) be the Lozinski measure of the matrix *B* with respect to the norm ∣ ∣ . ∣ ∣ in *R* ^*n*^ (see [20]) defined by

13 $$ \ell (B)=\underset{x\to 0}{\lim}\frac{\mid I+ Bx\mid -1}{x}. $$

Hence, if $\overline{q}<0$, this shows that the presence of any orbit gives rise to a simple closed rectifiable curve, such as periodic orbits and heterocyclic cycles.

Lemma 3 *Let U be simply connected, and conditions 3 and 4 be satisfied, then the unique equilibrium x* ^∗^ *of eq.* (10) *is globally asymptotically stable in U, if* $\overline{q}<0$ *.*

Now we apply the above techniques to prove the global stability of model (1) at disease-free equilibrium and endemic equilibrium, respectively. Thus, we have the following stability results.

**Theorem 3** *If R*_0_ < 1*, the proposed model (1) is globally asymptotically stable at disease-free equilibrium E*_0_ *and unstable otherwise.*

*Proof*: Let *χ*_1_ = (*S*(*t*), *V*(*t*)) and *χ*_2_ = (*L*(*t*), *A*(*t*), *B*(*t*), *C*(*t*)) represent the number of uninfected and infected individuals, respectively. Define *E*_0_ = (*χ*_0_, 0), where

14 $$ {\chi}_1^0=\left(\frac{b\left(\varphi +{\xi \mu}_0\right)}{\mu_0\left({\mu}_0+v+\varphi \right)},\frac{b\left({\mu}_0+v-{\mu}_0\xi \right)}{\mu_0\left({\mu}_0+v+\varphi \right)}\right). $$

Now using the proposed model (1), we have

15 $$ {\displaystyle \begin{array}{ll}\frac{d{\chi}_1}{dt}\hfill & =G\left({\chi}_1,{\chi}_2\right),\hfill \\ {}\frac{d{\chi}_1}{dt}\hfill & =\left(\begin{array}{cc}\hfill w(t)\hfill & \hfill \hfill \\ {}\hfill b\left(1-\xi \right)-\left({\mu}_0+\varphi \right)V(t)+ vS(t)\hfill & \hfill \hfill \end{array}\right),\hfill \end{array}} $$

where *w*(*t*) = *bξ*(1 − *ηC*(*t*)) + *φV*(*t*) − (*βA*(*t*) + *γβB*(*t*) + *ζβB*(*t*))*S*(*t*) − (*μ*_0_ + *v*)*S*(*t*). Thus, for *S* = *S*_0_, *V* = *V*_0_, and *G*(*χ*_1_, 0) = 0, eq. (11) becomes

16 $$ G\left({\chi}_1,0\right)=\left(\begin{array}{cc}\hfill b\xi +\varphi {V}_0-\left({\mu}_0+v\right){S}_0\hfill & \hfill \hfill \\ {}\hfill b\left(1-\xi \right)+{vS}_0-\left({\mu}_0+\varphi \right){V}_0\hfill & \hfill \hfill \end{array}\right). $$

Thus, from eq. (16) as *t* → ∞, ${\chi}_1\to {\chi}_1^0$. Thus, ${\chi}_1={\chi}_1^0$ is globally asymptotically stable.

Now to prove the second condition, that is $H\left({\chi}_1,{\chi}_2\right)=B{\chi}_1-\overline{H}\left({\chi}_1,{\chi}_2\right)$, we have

17 $$ B{\chi}_1-\overline{H}\left({\chi}_1,{\chi}_2\right)=\left(\begin{array}{cccc}\hfill -{c}_{11}\hfill & \hfill \beta {S}_0\hfill & \hfill \gamma \beta {S}_0\hfill & \hfill \zeta \beta {S}_0\hfill \\ {}\hfill \sigma \hfill & \hfill -{c}_{22}\hfill & \hfill 0\hfill & \hfill 0\hfill \\ {}\hfill 0\hfill & \hfill {c}_{32}\hfill & \hfill -{c}_{33}\hfill & \hfill 0\hfill \\ {}\hfill 0\hfill & \hfill p{\gamma}_1\hfill & \hfill 0\hfill & \hfill -{c}_{44}\hfill \end{array}\right)\left(\begin{array}{c}\hfill L(t)\hfill \\ {}\hfill A(t)\hfill \\ {}\hfill B(t)\hfill \\ {}\hfill C(t)\hfill \end{array}\right)-\left(\begin{array}{c}\hfill \varpi (t)\hfill \\ {}\hfill 0\hfill \\ {}\hfill 0\hfill \\ {}\hfill 0\hfill \end{array}\right), $$

where *c*_11_ = *μ*_0_ + *σ*, *c*_22_ = *μ*_0_ + *γ*_1_ + *ψ*, *c*_32_ = (1 − *p*)*γ*_1_, *c*_33_ = (*μ*_0_ + *μ*_1_ + *γ*_2_), *c*_44_ = *μ*_0_ + *μ*_2_ + *γ*_3_ + *bηξ*, and *ϖ*(*t*) = *βS*_0_ *L*(*t*) + *γβS*_0_ *A*(*t*) + *ζβS*_0_ *C*(*t*) − (*βSL*(*t*) + *γβSA*(*t*) + *ζβSC*(*t*)). Thus, matrix *B* and $\overline{H}\left({\chi}_1,{\chi}_2\right)$ are given by

18 $$ B=\left(\begin{array}{cccc}\hfill -{c}_{11}\hfill & \hfill \beta {S}_0\hfill & \hfill \gamma \beta {S}_0\hfill & \hfill \zeta \beta {S}_0\hfill \\ {}\hfill \sigma \hfill & \hfill -{c}_{22}\hfill & \hfill 0\hfill & \hfill 0\hfill \\ {}\hfill 0\hfill & \hfill {c}_{32}\hfill & \hfill -{c}_{33}\hfill & \hfill 0\hfill \\ {}\hfill 0\hfill & \hfill p{\gamma}_1\hfill & \hfill 0\hfill & \hfill -{c}_{44}\hfill \end{array}\right),\kern1em \overline{H}\left({\chi}_1,{\chi}_2\right)=\left(\begin{array}{c}\hfill \varpi (t)\hfill \\ {}\hfill 0\hfill \\ {}\hfill 0\hfill \\ {}\hfill 0\hfill \end{array}\right). $$

From the model (1), the total population is bounded by *S*_0_, that is, *S*, *L*, *A*, *B*, *C* ≤ *S*_0_, so *βSL* ≤ *βS*_0_ *I*, *βSA* ≤ *βS*_0_ *A*, *βSB* ≤ *βS*_0_ *B*, and *βSC* ≤ *βS*_0_ *C*, which implies that $\overline{H}\left({\chi}_1,{\chi}_2\right)$ is positive definite. In addition, from eq. (18), it is clear that matrix *B* is an M-matrix; that is, the off-diagonal elements are non-negative. Thus, conditions 1 and 2 are satisfied, so by Lemma 1, the disease-free equilibrium point *E*_0_ is globally asymptotically stable.

**Theorem 4** *If R*_0_ > 1*, the model (1) is globally asymptotically stable at endemic equilibrium E*_1_ *and unstable otherwise.*

*Proof*: To prove the global asymptotic stability of the proposed model (1) at endemic equilibrium *E*_1_, let us consider the subsystem of (1), such that

19 $$ {\displaystyle \begin{array}{ll}\frac{dS(t)}{dt}\hfill & = b\xi \left(1-\eta C(t)\right)+\varphi V(t)-\beta \left(A(t)+\gamma \beta B(t)+\zeta \beta C(t)\right)S(t)\hfill \\ {}\hfill & -\left({\mu}_0+v\right)S(t),\hfill \\ {}\frac{dL(t)}{dt}\hfill & =\beta S(t)A(t)+\gamma \beta S(t)B(t)+\zeta \beta S(t)C(t)-\left(\sigma +{\mu}_0\right)L(t),\hfill \\ {}\frac{dA(t)}{dt}\hfill & =\sigma L(t)-\left({\mu}_0+{\gamma}_1+\psi \right)A(t).\hfill \end{array}} $$

Obviously, the endemic equilibrium point *E*_1_ of the system (1) is locally asymptotically stable. Let *J*_2_ be the variational matrix of the system (19) given by

20 $$ {J}_2=\left(\begin{array}{ccc}\hfill -{j}_{11}\hfill & \hfill 0\hfill & \hfill -\beta S\hfill \\ {}\hfill {j}_{21}\hfill & \hfill -{j}_{22}\hfill & \hfill \beta S\hfill \\ {}\hfill 0\hfill & \hfill \sigma \hfill & \hfill -{j}_{33}\hfill \end{array}\right), $$

where

$$ {\displaystyle \begin{array}{ll}{j}_{11}\hfill & =\beta A(t)+\gamma \beta B(t)+\zeta \beta C(t)+2{\mu}_0+v+\sigma, \hfill \\ {}{j}_{22}\hfill & =\beta A(t)+\gamma \beta B(t)+\zeta \beta C(t)+2{\mu}_0+v+{\gamma}_1+\psi, \hfill \\ {}{j}_{32}\hfill & =\beta A(t)+\gamma \beta B(t)+\zeta \beta C(t),\hfill \\ {}{j}_{33}\hfill & =2{\mu}_0+\sigma +{\gamma}_1+\psi .\hfill \end{array}} $$

The second additive compound matrix of *J*_2_ is denoted by ${J}_2^{\mid 2\mid },$ which becomes

21 $$ {J}_2^{\mid 2\mid }=\left(\begin{array}{ccc}\hfill -\left({j}_{11}+{j}_{22}\right)\hfill & \hfill \beta S\hfill & \hfill \beta S\hfill \\ {}\hfill \sigma \hfill & \hfill -\left({j}_{11}+{j}_{33}\right)\hfill & \hfill 0\hfill \\ {}\hfill 0\hfill & \hfill {j}_{21}\hfill & \hfill -\left({j}_{22}+{j}_{33}\right)\hfill \end{array}\right). $$

Now choose a function $P\left(\chi \right)=P\left(S,L,A\right)=\mathit{\operatorname{diag}}\left\{\frac{S}{L},\frac{S}{L},\frac{S}{L}\right\},$ which implies that ${P}^{-1}\left(\chi \right)=\mathit{\operatorname{diag}}\left\{\frac{L}{S},\frac{L}{S},\frac{L}{S}\right\},$ Then, taking the time derivative, that is, *P*_*f*_(*χ*), we obtain

22 $$ {P}_f\left(\chi \right)=\mathit{\operatorname{diag}}\left\{\frac{\dot{S}}{S}-\frac{S\dot{L}}{L^2},\frac{\dot{S}}{S}-\frac{S\dot{L}}{L^2},\frac{\dot{S}}{S}-\frac{S\dot{L}}{L^2}\right\}. $$

Now ${P}_f{P}^{-1}=\mathit{\operatorname{diag}}\left\{\frac{\dot{S}}{S}-\frac{\dot{L}}{L},\frac{\dot{S}}{S}-\frac{\dot{L}}{L},\frac{\dot{S}}{S}-\frac{\dot{L}}{L}\right\}$ and *PJ* ^∣2∣^ *P* ^−1^ = *J* ^∣2∣^. Thus, we take *B* = *P*_*f*_ *P* ^−1^ + *PJ* ^∣2∣^ *P* ^−1^, which can be written as

23 $$ B=\left(\begin{array}{cc}\hfill {B}_{11}\hfill & \hfill {B}_{12}\hfill \\ {}\hfill {B}_{21}\hfill & \hfill {B}_{22}\hfill \end{array}\right), $$

where

$$ {\displaystyle \begin{array}{ll}{B}_{11}\hfill & =-\frac{\dot{S}}{S}-\frac{\dot{L}}{L}-\beta A(t)-\gamma \beta B(t)-\zeta BC(t)-2{\mu}_0-v-\sigma, \hfill \\ {}{B}_{12}\hfill & =\left(\beta S(t)\kern0.5em \beta S(t)\right),\hfill \\ {}{B}_{21}\hfill & =\left(\begin{array}{c}\hfill \sigma \hfill \\ {}\hfill 0\hfill \end{array}\right),\hfill \\ {}{B}_{22}\hfill & =\left(\begin{array}{cc}\hfill {x}_{11}\hfill & \hfill 0\hfill \\ {}\hfill {x}_{21}\hfill & \hfill {x}_{22}\hfill \end{array}\right),\hfill \end{array}} $$

with ${x}_{11}=\frac{\dot{S}}{S}-\frac{\dot{L}}{L}-\beta A(t)-\gamma \beta B(t)-\zeta \beta C(t)-2{\mu}_0-v-{\gamma}_1-\psi,$ *x*_21_ = *βA*(*t*) + *γβB*(*t*) + *ζβC*(*t*), and ${x}_{22}=\frac{\dot{S}}{S}-\frac{\dot{L}}{L}-2{\mu}_0-\sigma -{\gamma}_1-\psi .$ Let (*a*_1_, *a*_2_, *a*_3_) be a vector in *R* ^3^ and its norm ∣ ∣ . ∣ ∣ defined by

24 $$ \mid \mid {a}_1,{a}_2,{a}_3\mid \mid =\max \left\{||{a}_1||,||{a}_2||+||{a}_3||\right\}. $$

Let *ℓ*(*B*) be the Lozinski measure with respect to the above norm described by Martin [20], then we choose

25 $$ \ell (B)\le \sup \left\{{g}_1,{g}_2\right\}=\sup \left\{\ell \left({B}_{11}\right)+||{B}_{12}||,\ell \left({B}_{22}\right)+||{B}_{21}||\right\}, $$

where ∣ ∣ *B*_12_ ∣ ∣ and ∣ ∣ *B*_21_ ∣ ∣ are matrix norms, then

26 $$ {g}_1=\ell \left({B}_{11}\right)+\mid \mid {B}_{12}\mid \mid, \kern1em {g}_2=\ell \left({B}_{22}\right)+\mid \mid {B}_{21}\mid \mid, $$

where $\ell \left({B}_{11}\right)=-\frac{\dot{S}}{S}-\frac{\dot{L}}{L}-\beta A(t)-\gamma \beta B(t)-\zeta BC(t)-2{\mu}_0-v-\sigma$, ∣ ∣ *B*_12_ ∣  ∣  = *βS*(*t*), $\ell \left({B}_{22}\right)=\max \left\{\frac{\dot{S}}{S}-\frac{\dot{L}}{L}-2{\mu}_0-v-{\gamma}_1-\psi, \frac{\dot{S}}{S}-\frac{\dot{L}}{L}-2{\mu}_0-\sigma -{\gamma}_1-\psi \right\}$ $=\frac{\dot{S}}{S}-\frac{\dot{L}}{L}-2{\mu}_0-{\gamma}_1-\psi -\min \left\{v,\sigma \right\}$ and ∣ ∣ *B*_21_ ∣  ∣  = max {*σ*, 0} = *σ*. Therefore, *g*_1_ and *g*_2_ becomes

$$ {\displaystyle \begin{array}{ll}{g}_1\hfill & =\frac{\dot{S}}{S}-\frac{\dot{L}}{L}-\beta A(t)-\gamma \beta B(t)-\zeta \beta C(t)-2{\mu}_0-\min \left\{v,\sigma \right\}+\beta S(t),\hfill \\ {}{g}_2\hfill & =\frac{\dot{S}}{S}-\frac{\dot{L}}{L}-2{\mu}_0-\sigma -{\gamma}_1-\psi, \hfill \end{array}} $$

which implies that

27 $$ {\displaystyle \begin{array}{ll}{g}_1\hfill & \le \frac{\dot{S}}{S}-2{\mu}_0-\min \left\{v,\sigma \right\},\hfill \\ {}{g}_2\hfill & \le \frac{\dot{S}}{S}-2{\mu}_0-{\gamma}_1-\psi .\hfill \end{array}} $$

Using eq. (27) in eq. (25), we obtain

28 $$ {\displaystyle \begin{array}{ll}\ell (B)\hfill & \le \sup \left\{{g}_1,{g}_2\right\}\le \left\{\frac{\dot{S}}{S}-2{\mu}_0-\min \left\{v,\sigma \right\},\frac{\dot{S}}{S}-2{\mu}_0-{\gamma}_1-\psi \right\},\hfill \\ {}\ell (B)\hfill & \le \left\{\frac{\dot{S}}{S}-2{\mu}_0-\min \Big\{\min \left\{v,\sigma \right\},{\gamma}_1-\psi \Big\}\right\}.\hfill \end{array}} $$

Hence, $\ell (B)\le \frac{\dot{S}}{S}-2{\mu}_0.$ Now integrating the Lozinski measure *ℓ*(*B*) with respect to *t* in the interval [0, *t*] and taking lim_*t* → ∞_, we obtain

29 $$ \underset{t\to \infty }{\lim}\sup \kern0.1em \sup \frac{1}{t}{\int}_0^t\ell (B)\kern0.1em dt<-2{\mu}_0<0. $$

From eq. (29), we have

30 $$ \overline{q}=\underset{t\to \infty }{\lim}\sup \kern0.1em \sup \frac{1}{t}{\int}_0^t\ell (B)\kern0.1em dt<0. $$

Thus, the system containing the first three equations of the model (1) is globally asymptotically stable around its interior equilibrium (*S*_1_, *L*_1_, *A*_1_). Now consider the subsystem of the model (1), such that

31 $$ {\displaystyle \begin{array}{ll}\frac{dB(t)}{dt}\hfill & =p{\gamma}_1A(t)-\left({\mu}_0+{\mu}_1+{\gamma}_2\right)B(t),\hfill \\ {}\frac{dC(t)}{dt}\hfill & = b\xi \eta C(t)+\left(1-p\right){\gamma}_1A(t)-\left({\mu}_0+{\mu}_2+{\gamma}_3\right)C(t),\hfill \\ {}\frac{dR(t)}{dt}\hfill & =\psi A(t)+{\gamma}_2B(t)+{\gamma}_3C(t)-{\mu}_0R(t),\hfill \\ {}\frac{dV(t)}{dt}\hfill & =b\left(1-\xi \right)+ vS(t)-\left({\mu}_0+\varphi \right)V(t).\hfill \end{array}} $$

By taking the limit of the system (31), we obtain

32 $$ {\displaystyle \begin{array}{ll}\frac{dB(t)}{dt}\hfill & =p{\gamma}_1{A}_1-\left({\mu}_0+{\mu}_1+{\gamma}_2\right)B(t),\hfill \\ {}\frac{dC(t)}{dt}\hfill & = b\xi \eta C(t)+\left(1-p\right){\gamma}_1{A}_1-\left({\mu}_0+{\mu}_2+{\gamma}_3\right)C(t),\hfill \\ {}\frac{dR(t)}{dt}\hfill & =\psi {A}_1+{\gamma}_2{B}_1+{\gamma}_3{C}_1-{\mu}_0R(t),\hfill \\ {}\frac{dV(t)}{dt}\hfill & =b\left(1-\xi \right)+{vS}_1-\left({\mu}_0+\varphi \right)V(t).\hfill \end{array}} $$

This solves the system (32) using the initial conditions *B*(0), *C*(0), *R*(0), and *V*(0). Thus, for large time *t*, that is, *t* → ∞, *B*(*t*) → *B*_1_, *C*(*t*) → *C*_1_, *R*(*t*) → *R*_1_, and *V*(*t*) → *V*_1_, which is sufficient to prove that the endemic equilibrium point *E*_1_ is globally asymptotically stable.

## Results and discussions

### Numerical results and discussion

In this section, the numerical simulations of the proposed model (1) are presented. The numerical results are obtained by using the fourth-order Runge–Kutta scheme [9, 10]. The simulation of our paper should be considered from a qualitative point of view, but not from the quantitative point of view. Therefore, for this purpose, some of the parameters are taken from published articles and some are assumed with feasible values. For our simulation, we consider the parameter values as follows: *b* = 0.0121, *ξ* = 0.8, *η* = 0.11, *β* = 0.012, *γ* = 0.46, *ζ* = 0.0123, *σ* = 0.0012, *φ* = 0.01, *ψ* = 0.012, *v* = 0.6, *p* = 0.6, *γ*_1_ = 0.33, *γ*_2_ = 0.009, *γ*_3_ = 0.025, *μ*_0_ = 0.069, *μ*_1_ = 0.000532, and *μ*_2_ = 0.000532. Some of these parameters, the birth rate *b*, natural death rate *μ*_0_, and proportion of perinatally infected individuals *η*, are taken from [13, 21, 22] and the remaining parameters are assumed with biologically feasible values.

**Fig. 1:**
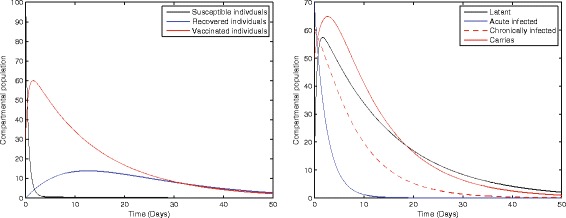
Solution curves of model (1) with respect to the following parameter values and initial size of the compartmental population *b* = 0.0121, *ξ* = 0.8, *η* = 0.11, *β* = 0.012, *γ* = 0.46, *ζ* = 0.0123, *σ* = 0.0012, *φ* = 0.01, *ψ* = 0.012, *v* = 0.6, *p* = 0.6, *γ*_1_ = 0.33, *γ*_2_ = 0.009, *γ*_3_ = 0.025, *μ*_0_ = 0.069, *μ*_1_ = 0.000532, *μ*_2_ = 0.000532, *S*(0) = 100, *A*(0) = 70, *B*(0) = 60, *C*(0) = 50, *R*(0) = 0, and *V*(0) = 30.

Fig. 1 represents the dynamical behavior of susceptible, recovered, vaccinated, latent, acute infected, chronically infected, and vaccinated individuals, respectively. Moreover, the time interval is taken 0–50, while the initial population size for the compartmental population susceptible, latent, acute infected, chronically infected, carriers, recovered, and vaccinated individuals are taken to be 100, 10, 70, 60, 50, 0, and 30, respectively. The simulation of our proposed model shows that the susceptible, acute infected, and chronically infected individuals decrease sharply, while the latent, carrier recovered, and vaccinated increase at the beginning and then decrease, as shown in Fig. 1.

### Sensitivity analysis

**Fig. 2:**
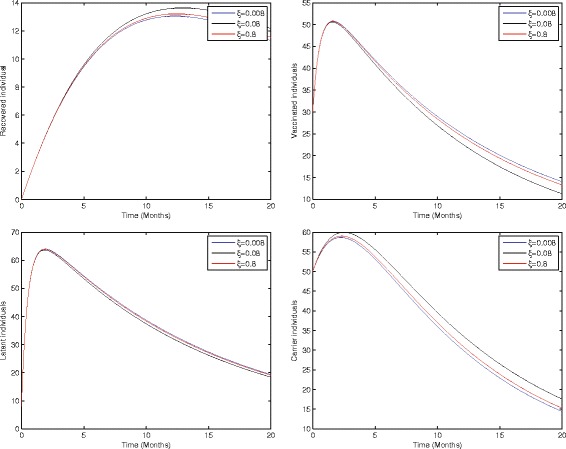
Sensitivity analysis of model (1) by varying the value of *ξ* = 0.008, 0.08, 0.8 with all other values fixed: *b* = 0.0121, *η* = 0.11, *β* = 0.012, *γ* = 0.46, *ζ* = 0.0123, *σ* = 0.0012, *φ* = 0.01, *ψ* = 0.012, *v* = 0.6, *p* = 0.6, *γ*_1_ = 0.33, *γ*_2_ = 0.009, *γ*_3_ = 0.025, *μ*_0_ = 0.069, *μ*_1_ = 0.000532, *μ*_2_ = 0.000532, *S*(0) = 100, *A*(0) = 70, *B*(0) = 60, *C*(0) = 50, *R*(0) = 0, and *V*(0) = 30.

**Fig. 3:**
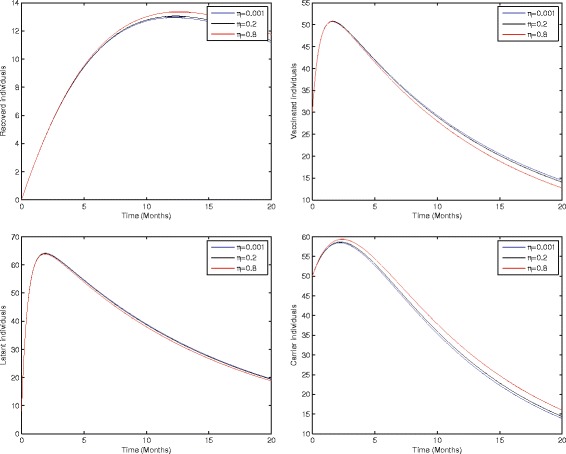
Sensitivity analysis of model (1) by varying the value of *η* = 0.001, 0.2, 0.8 with all other parameters fixed: *b* = 0.0121, *ξ* = 0.8 *β* = 0.012, *γ* = 0.46, *ζ* = 0.0123, *σ* = 0.0012, *φ* = 0.01, *ψ* = 0.012, *v* = 0.6, *p* = 0.6, *γ*_1_ = 0.33, *γ*_2_ = 0.009, *γ*_3_ = 0.025, *μ*_0_ = 0.069, *μ*_1_ = 0.000532, *μ*_2_ = 0.000532, *S*(0) = 100, *A*(0) = 70, *B*(0) = 60, *C*(0) = 50, *R*(0) = 0, and *V*(0) = 30.

**Fig. 4:**
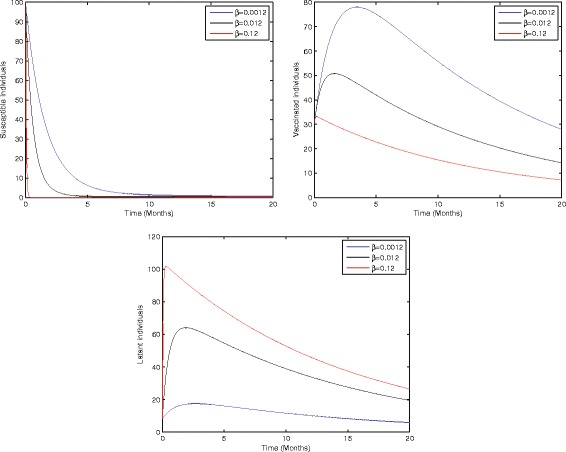
Sensitivity analysis of model (1) by varying the value of *β* = 0.0012, 0.012, 0.12 with all other parameters fixed: *b* = 0.0121, *η* = 0.8, *ξ* = 0.8, *γ* = 0.46, *ζ* = 0.0123, *σ* = 0.0012, *φ* = 0.01, *ψ* = 0.012, *v* = 0.6, *p* = 0.6, *γ*_1_ = 0.33, *γ*_2_ = 0.009, *γ*_3_ = 0.025, *μ*_0_ = 0.069, *μ*_1_ = 0.000532, *μ*_2_ = 0.000532, *S*(0) = 100, *A*(0) = 70, *B*(0) = 60, *C*(0) = 50, *R*(0) = 0, and *V*(0) = 30.

**Fig. 5:**
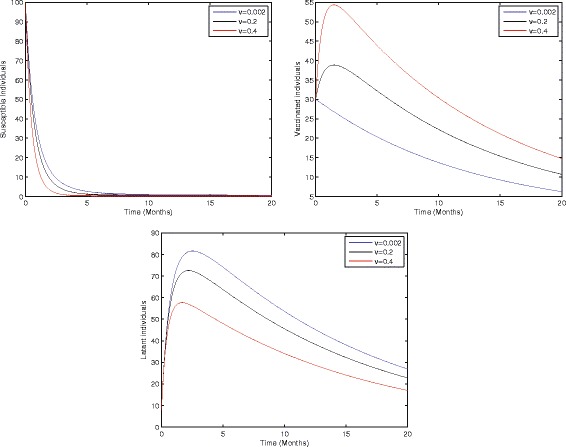
Sensitivity analysis of model (1) by varying the value of *v* = 0.002, 0.2, 0.4 with all other parameters fixed: *b* = 0.0121, *ξ* = 0.8, *η* = 0.8, *β* = 0.012, *γ* = 0.46, *ζ* = 0.0123, *σ* = 0.0012, *φ* = 0.01, *ψ* = 0.012, *p* = 0.6, *γ*_1_ = 0.33, *γ*_2_ = 0.009, *γ*_3_ = 0.025, *μ*_0_ = 0.069, *μ*_1_ = 0.000532, *μ*_2_ = 0.000532, *S*(0) = 100, *A*(0) = 70, *B*(0) = 60, *C*(0) = 50, *R*(0) = 0, and *V*(0) = 30.

In the study of biological dynamics, the transmission dynamics of infectious disease sensitivity analysis play an especially important role. Using sensitivity analysis, we can investigate the role of each parameter used in the model and can easily develop a strategy to control the spread of infection in the community. To do this, local sensitivity analysis of the proposed model (1) has been carried out by varying parameters such as the birth rate, birth rate without successful vaccination, proportion of perinatally infected individuals, interaction rate of infected and susceptible individuals, vaccination, and the average probability of those individuals who fail to recover in acute stage and develop the chronic stage. Thus, Figs 2–5 represents the sensitivity analysis of our proposed model (1) with respect to birth rate without successful vaccination, proportion of perinatally infected individuals, the interaction rate of susceptible and infected individuals, and vaccination.

Figure 2 shows that the birth rate without successful vaccination is directly proportional to carrier and inversely proportional to susceptible and vaccinated individuals, while having no impact on acute and chronically infected individuals, which shows that the inflow of newborns without successful vaccination will increase the risk of carrier individuals. Fig. 3 represents that the rate of perinatally infected individuals is directly proportional to carrier and inversely proportional to latent and vaccinated individuals, while it has no impact on susceptible, acute infected, or and chronically infected individuals. Similarly to the inflow of newborns without successful vaccination, perinatally infected individuals will also increase the risk of the carrier population. Fig. 4 shows that the transmission/contact rate is directly proportional to the number of infected individuals including the latent, acute infected, chronically infected, and carrier individuals, while inversely proportional susceptible, recovered, and vaccinated individuals, which shows that the increasing contact rate of infected and non-infected will increase the risk of the infected population. Fig. 5 shows that the vaccination rate is directly proportional to recovered and vaccinated individuals and inversely proportional to susceptible and latent individuals, which illustrates that increasing vaccination will decrease the risk of an infected population. Thus, from the above discussion it is clear that for the control of hepatitis B, we need to pay more attention to the above risk factors.

## Conclusion

In this article, we established a model for the transmission dynamics of hepatitis B by taking into account the classification of different phases of individuals infected with hepatitis B. We studied different mathematical analyses, including equilibrium analysis and boundedness, and obtained the basic reproduction number by using the next-generation matrix. Moreover, we discussed the stability analysis and showed that the established model is both locally as well as globally asymptotically stable for the possible equilibria. To discuss the local stability, linearization and Routh—Herwitz criteria were used, while global stability was retrieved by using the method of Castillo-Chávez et al. and a geometrical approach. Finally, the numerical simulation and sensitivity analysis were presented to show the feasibility of the proposed work. Our work provides a coherent platform for studying the full dynamics of hepatitis B and an effective direction for theoretical work. The techniques used in this article are also applicable to other epidemic models.
